# Show Me My Health Plans: a study protocol of a randomized trial testing a decision support tool for the federal health insurance marketplace in Missouri

**DOI:** 10.1186/s12913-016-1314-9

**Published:** 2016-02-16

**Authors:** Mary C. Politi, Abigail R. Barker, Kimberly A. Kaphingst, Timothy McBride, Enbal Shacham, Carey S. Kebodeaux

**Affiliations:** Division of Public Health Sciences, Department of Surgery, Washington University in St. Louis School of Medicine, St. Louis, USA; George Warren Brown School of Social Work, Washington University in St. Louis, St. Louis, USA; Department of Communication, Huntsman Cancer Institute, University of Utah, Salt Lake City, USA; College for Public Health and Social Justice, Saint Louis University, Saint Louis, USA

**Keywords:** Health insurance, Health literacy, Decision aids, Decision support, Health communication

## Abstract

**Background:**

The implementation of the ACA has improved access to quality health insurance, a necessary first step to improving health outcomes. However, access must be supplemented by education to help individuals make informed choices for plans that meet their individual financial and health needs.

**Methods/Design:**

Drawing on a model of information processing and on prior research, we developed a health insurance decision support tool called *Show Me My Health Plans*. Developed with extensive stakeholder input, the current tool (1) simplifies information through plain language and graphics in an educational component; (2) assesses and reviews knowledge interactively to ensure comprehension of key material; (3) incorporates individual and/or family health status to personalize out-of-pocket cost estimates; (4) assesses preferences for plan features; and (5) helps individuals weigh information appropriate to their interests and needs through a summary page with “good fit” plans generated from a tailored algorithm. The current study will evaluate whether the online decision support tool improves health insurance decisions compared to a usual care condition (the healthcare.gov marketplace website). The trial will include 362 individuals (181 in each group) from rural, suburban, and urban settings within a 90 mile radius around St. Louis. Eligibility criteria includes English-speaking individuals 18–64 years old who are eligible for the ACA marketplace plans. They will be computer randomized to view the intervention or usual care condition.

**Discussion:**

Presenting individuals with options that they can understand tailored to their needs and preferences could help improve decision quality. By helping individuals narrow down the complexity of health insurance plan options, decision support tools such as this one could prepare individuals to better navigate enrollment in a plan that meets their individual needs. The randomized trial was registered in clinicaltrials.gov (NCT02522624) on August 6, 2015.

## Background

Since the implementation of the Affordable Care Act (ACA), approximately 7 million individuals have enrolled in the ACA marketplace [1]. Providing access to quality health insurance is a necessary first step to improving health outcomes among the previously uninsured or underinsured. However, access needs to be supplemented by education about how to select a plan that best meets one’s individual financial and health needs.

The majority of individuals who enroll in the marketplace struggle to make sense of the complex information necessary to make an informed plan choice. Most individuals have limited health insurance literacy, and only about 24 % of individuals feel confident that they understand health insurance concepts and terms [2, 3]. As a result, in one study examining marketplace enrollment, only 50 % of consumers chose a plan that offered acceptable coverage for their health status [4]. Recent data demonstrates that almost a quarter of individuals who enrolled in the ACA marketplace chose high deductible plans without a health savings account [5]. Given that the population of individuals served by the ACA marketplace is disproportionately low-income, high deductible plans could leave them with enormous out-of-pocket expenses that they are unable to pay. Our own research suggests that individuals attend primarily to insurance premiums and do not always consider or understand the importance of deductibles, formularies, and out-of-pocket maximums in making their choices [6, 7].

Understanding insurance plan information is more challenging for those with limited health literacy and numeracy skills given the numeric calculations required to estimate out-of-pocket costs across plans [1]. Consumers find it difficult to decipher the language and compare values in traditional health plan summaries [8]. Our formative work showed that individuals with limited health literacy and numeracy skills have difficulty interpreting information presented in insurance plans even when presented in plain language summaries of this information [9]. When individuals do not understand information, they are more likely to ignore it [10] even when it is critical to their decision.

Information processing models suggest that individuals can only process information using a limited number of variables at any given time before they experience cognitive overload, especially in new situations in which they are faced with complex and unfamiliar information [11]. In addition, individuals are often forced to make trade-offs among factors (e.g., cost of a premium vs. cost of a deductible) as they sort through their choices. When trade-offs are difficult to make, individuals often take mental short-cuts to facilitate decision making, such as choosing one single factor to dominate the decision (e.g., premium cost), even if that short-cut is not in the individual’s best interest [12].

**Fig. 1:**
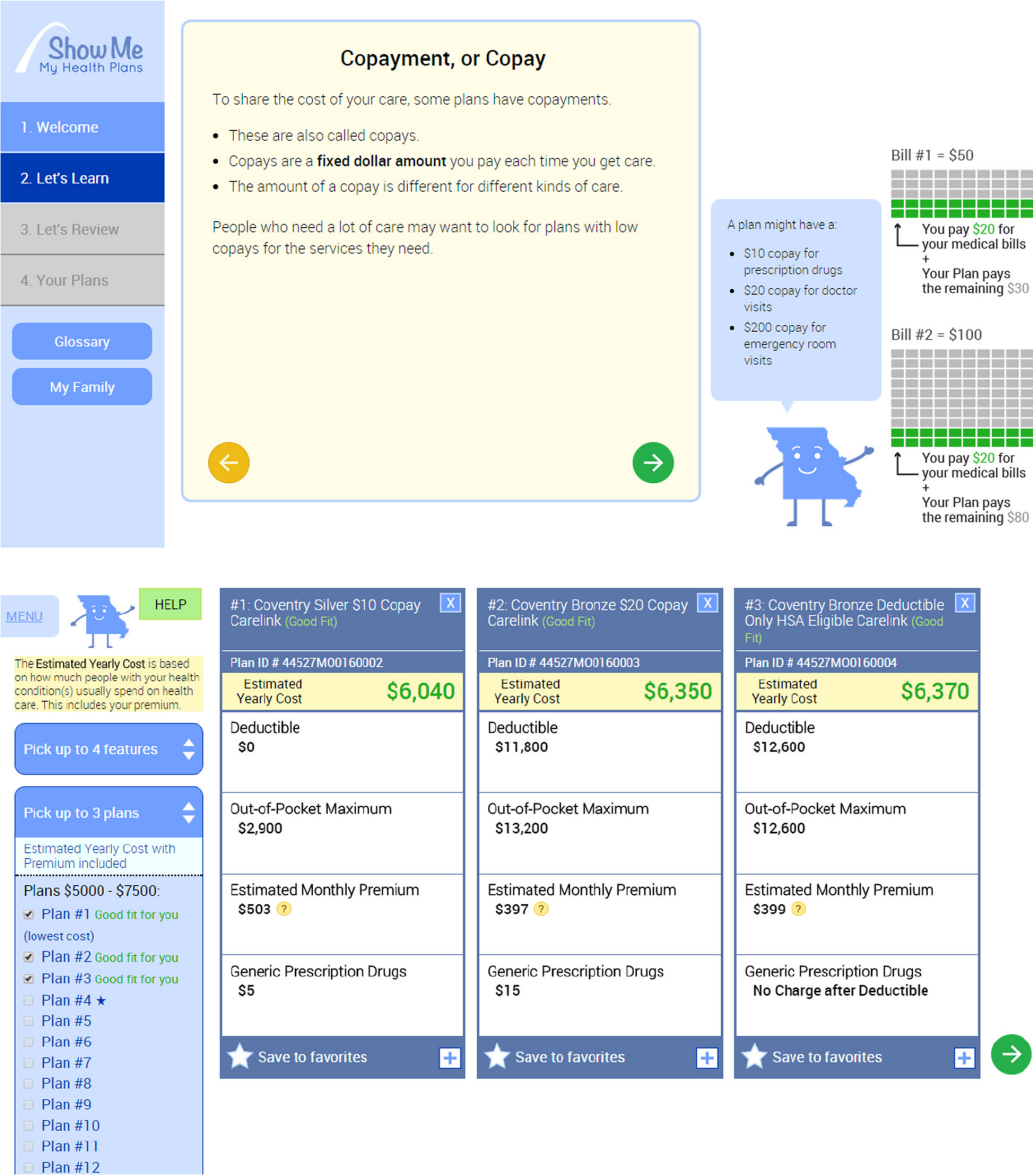
Screenshots from *Show Me My Health Plans.* Our final card sorting feature uses an algorithm to predict annual expenditures based on MEPS data and the plan details in the ACA marketplace plans available in their county. It also shows them features of the top plans that are personalized to their annual expenditures plus preferences. They can save plans to favorites and see all plans available to them if they want to trade off more coverage at a higher cost, for example, but our algorithm sorts them by annual plan cost to them/their families (also factoring in potential financial risks based on number of health conditions and the probability that they might incur very high costs associated with them in any given calendar year)

A recent report from the Robert Wood Johnson Foundation [*Plan Choice Decision Support* [13] outlines current health plan decision support needs, as well as solutions and important innovation breakthroughs. The report calls for the development of a health insurance decision support tool and highlights three critical components this tool would need to include: 1) estimating yearly out-of-pocket costs for users; 2) eliciting user needs and preferences; and 3) guiding users to plan options that match their use and preferences. In this study, we developed a health insurance decision support tool for individuals living in Missouri enrolling in the federal marketplace. Our tool is based on a model of information processing and empirical research on decision-making [14] and addresses each of the critical components of health insurance decision needs specified in the Robert Wood Johnson report. Our health insurance decision support tool called *Show Me My Health Plans* [see Fig. 1]:

1. Simplifies information to reduce the cognitive burden on individuals by using plain language and graphics, and it provides a step-by-step process for thinking about health insurance;
2. Assesses knowledge and provides interactive feedback to users to ensure comprehension of key information;
3. Makes information relevant to the user by asking them about their age, county of residence, current health status, family members’ ages and health status if covering family members, and estimates out-of-pocket costs across plans based on national estimates of how much care individuals use across various ages and conditions;
4. Assesses preferences for insurance features;
5. Helps individuals use and weigh information appropriate to their interests and needs by creating a summary page that displays health insurance plan information for their county by estimated annual out-of-pocket costs. It also displays three “good fit” plans based on their preferences, health status, and the annual estimated out-of-pocket costs.

This manuscript describes the process for developing this health insurance decision support tool and our study protocol for our randomized trial evaluating the tool during the fall open enrollment. The randomized trial is registered in clinicaltrials.gov (NCT02522624).

## Methods

Our earlier work tested ways to present information to uninsured individuals who were eligible for the ACA exchange plans. Our initial paper-based strategies included 1) a plain language table, “layering” information with basic facts preceding more specific details and using language from individuals’ own words from our qualitative study; 2) a visual condition helping individuals to distinguish among plan details and to process information in smaller pieces without looking at it all at once; and 3) a narrative condition enhancing relevance to users, helping them identify specific details that matter most to them through personal stories. We tested these strategies in a randomized experiment, using three hypothetical plans based on the MA Commonwealth plans (since we developed the initial strategies before the ACA exchanges were released). In all three conditions, participants made value-consistent choices, choosing plans that matched their stated preferences for insurance features. Those with higher health literacy skills were more knowledgeable and had a preference for the preference for plain language table over other conditions. Those with lower health literacy skills showed no preference for study condition [9].

In the current ongoing study, to develop the tool, we (1) engaged stakeholders to determine how to modify the previous decision support strategies; (2) programmed the successful elements from our strategies (e.g., plain language summaries, with new graphics responding to stakeholder feedback) into an tool online, removing narrative information based on stakeholder feedback and our earlier data and undergoing an iterative process for editing language based on principles of health literacy and health communication; (3) incorporated individual and/or family health status to personalize estimated out-of-pocket costs across ACA marketplace plans; and (4) assessed user preferences for health insurance features. We are testing whether our decision tool and the algorithm that predicts which plans might be a good fit for individuals based on their health status and preferences improves health insurance decisions compared to usual care (the healthcare.gov marketplace website).

**Fig. 2:**
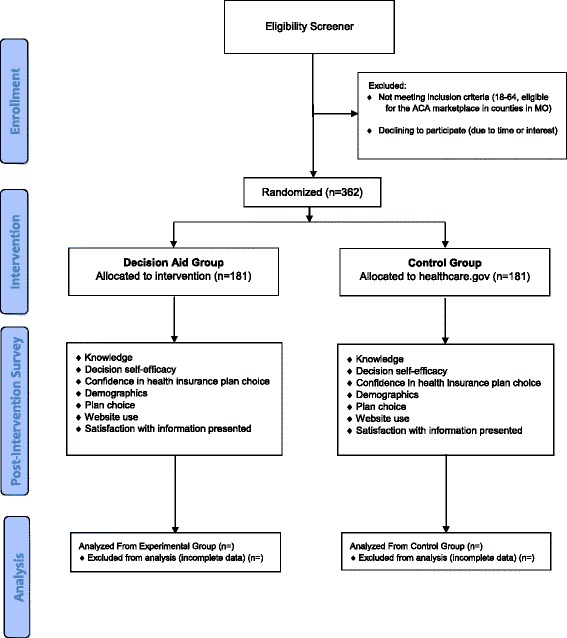
Planned study flow diagram

The trial will include 362 individuals (181 in each group, decision tool or usual care) from rural, suburban, and urban settings in MO counties within 90-miles of St. Louis. They will be computer randomized to view the intervention or usual care condition. Eligibility criteria include English speaking individuals 18–64 years old who are eligible for the ACA marketplace plans. Recruitment will occur between October 2015 and February 2016. Individuals enrolled prior to 2016 open enrollment (November 1, 2015) will view the 2015 plan data in both the intervention and usual care condition to help prepare them for future enrollment. The decision tool was updated when the 2016 data became available (November 1, 2015). Figure 2 shows a study flow chart; we will follow CONSORT guidelines for the design and reporting of results.

Participants will complete the study and survey online prior to enrolling either on their own or with a certified application counselor or insurance broker. Participation lasts approximately 30–40 min; there is no follow-up period. Participants will receive a $20 gift card as compensation for their time. The study was approved by the Human Research Protection Office (HRPO) at Washington University in St. Louis.

### Estimating annual out-of-pocket costs

In order to personalize estimated health spending per calendar year, we derived estimates from The Medical Expenditure Panel Survey (MEPS). MEPS began collecting data in 1996 and is a set of large-scale surveys of families and individuals, their medical providers, and employers across the U.S. MEPS collects data on the specific health services that Americans use, how frequently they use them, the cost of these services, and how they are paid for, as well as data on the cost, scope, and breadth of health insurance held by and available to workers. The MEPS Household Component provides data from individual households and their members, which is supplemented by data from their medical providers. It fields questionnaires to individual household members to collect nationally representative data on demographic characteristics, health conditions, health status, use of medical care services, charges and payments, access to care, satisfaction with care, health insurance coverage, income, and employment.

MEPS health conditions are fully-specified ICD-9-CM codes. They are determined through the interview process by probing respondents’ answers to questions in the categories of (1) condition enumeration, (2) medical events, and (3) disability days and are professionally coded. The MEPS design is one of overlapping panels, in which respondents participate for 2 years, but medical conditions files are available for each calendar year. They contain data on about 35,000 people per year, half of whom are new to the sample each year. Merging several years of data can easily generate expenditure data on 100,000 unique individuals.

We used MEPS data to estimate health spending per calendar year for those with either no medical conditions, or one or more of the following common or costly medical conditions: 1) Arthritis; 2) Musculoskeletal Conditions; 3) chronic obstructive pulmonary disease (COPD); 4) Asthma; 5) Cancer; 6) Diabetes; 7) Depression; 8) Anxiety; 9) Other Mental Illness; 10) Stroke; 11) Hypertension; 12) Heart Condition; 13) Epilepsy; 14) Attention Deficit Hyperactivity Disorder (ADHD). We also estimate spending by age and gender.

### Algorithm

The method for estimating an individual’s or family’s expected out-of-pocket costs has several steps. MEPS data are used to estimate a two-stage model because many individuals actually incur no costs at all during a calendar year. The model estimates first the probability of incurring any positive amount of costs, based upon the presence of the conditions as well as age and gender, and it then estimates the size of these costs based upon the same variables (and conditional upon incurring costs at all). The resulting value is called the “expected costs” for the individual, and it is compared against the specific details of each plan being assessed. For example, if the expected costs are below a plan’s deductible, we assume that the individual will be responsible for all of those costs. If the expected costs fall in between a plan’s deductible and its out-of-pocket maximum, we use additional MEPS data to estimate the likely mix of healthcare services the individual will utilize and then apply the plan’s cost-sharing information to obtain an estimate of the out-of-pocket costs to the individual. If the expected costs fall above the plan’s out-of-pocket maximum, then we assume that the dollar amount of the out-of-pocket maximum will be paid by the individual.

The above algorithm is fairly straightforward, but it does not, by itself, yield the best recommendations for health insurance plans. Health insurance is, by its nature, a product that does not benefit its consumers in terms of expected values or averages. Individuals benefit from health insurance mainly because it shields them from the risk of incurring expenses that they do not expect. Thus, it was necessary to add risk-adjustment factors to the algorithm in order to generate an appropriate set of recommendations for individuals with chronic conditions. This part of the algorithm sorts the plans pairwise, comparing them two at a time until it has ordered them all. For example, if we compare a more expensive plan with better coverage to a less expensive plan with less coverage, we will score the more expensive one more highly if it allows the individual to avoid a significant risk of being responsible for large medical bills while only paying a slightly higher premium. The way this risk is assessed depends upon the individual’s age, gender, and conditions, so individuals with a higher risk of large medical bills might be directed toward silver and gold plans as “best fit” plans, even when a simple comparison of expected costs might not support that recommendation initially. Individuals viewing the information will be able to see all plans sorted by lowest to highest annual cost, as well as three “good fit” plans based on this algorithmic prediction.

### ACA marketplace data

Our decision tool asks participants about their incomes, their county of residence, and the ages and tobacco use status of all family members for whom coverage is being sought. These are the factors which will affect the premiums charged. We use ACA Health Insurance Marketplace data on plans offered in Missouri by county, which specifies the premium of each plan as well as detailed cost sharing information (copays, coinsurance, deductibles, etc.). The file also contains information on the reduced cost sharing (lower copays, etc.) available to those whose incomes are near the Federal Poverty Level (FPL). All plans are scored by metal level (bronze, silver, gold, and platinum), with these levels being required to deliver a certain amount of value to the average consumer. Under reduced cost-sharing provisions, the required value is higher for those with incomes nearest the FPL, but only when they purchase silver plans. We use individuals’ income information to extract the relevant offerings and all associated parameters from the federal plan file.

Additionally, individuals with incomes between 100 % and 400 % FPL are eligible for subsidies on a sliding scale. The subsidy is based upon the price of the second-lowest silver plan being sold in that individual’s county: if the second-lowest silver plan costs more than a certain percentage of income, then whatever additional amount is needed to purchase that plan is the amount of the subsidy. That amount can be applied to the purchase of any plan, not just the second-lowest silver one. This cap on spending is applied at the family level.

Finally, the ACA allows for a tobacco surcharge on premiums of up to 50 %. Issuers vary on the degree to which they assess this surcharge and on how they implement it in families where some members smoke and others do not, so we manually obtained information on each firm participating in Missouri marketplaces and added it to the calculation. The tobacco surcharge is not included in subsidy calculations, i.e., the cap is determined by comparing the second-lowest silver no-tobacco premium to the family’s income.

### Outcomes

Our primary outcomes are knowledge, decision self-efficacy, and confidence in health insurance plan choice. To measure knowledge, we will use the health insurance knowledge questions we developed and tested in our earlier work [9]. These items were initially developed based on standard decision tool measures [15]. We developed items based on information that is essential to making a health plan decision (e.g., understanding key terms, or understanding facts that differentiate options), and then modified them with participant feedback and pilot testing. Response options include true/ false/ unsure. Total number of correct responses to the true/false/unsure items are calculated. Cronbach’s alpha for knowledge items in our earlier sample (*N* = 343) was 0.61.

To assess decision self-efficacy, we will use the lower literacy version of the Decision Self-Efficacy Scale [16], a validated measure of an individual’s self-confidence or belief in their ability to make a decision. Individuals are asked to rate on a three-item scale how confident they feel taking actions involved in making an informed choice (e.g., gathering information, asking questions, expressing opinions, seeking advice). This scale has been validated among individuals with schizophrenia and osteoporosis, and has been used to study health literacy and shared decision making among cancer patients, lower SES populations, and postmenopausal women, among others [16–19]. It has high levels of internal consistency (mean Cronbach’s alpha = 0.86) [16]. It is correlated with scales assessing feeling informed, supported, and knowledgeable about decisions [16].

To measure confidence in choice, we will use the lower literacy version of the validated Decisional Conflict Scale [20, 21] (DCS) to assess whether individuals feel they have enough information to make a choice, whether they are clear about which choice to make, and whether they felt confident in their ability to make a good decision. Studies have found the DCS to have strong reliability with alphas ranging from 0.78 to 0.96 [21–23]. Cronbach’s alpha for this measure in our earlier work studying uninsured participants (*N* = 343) was 0.74.

We will also measure choice, tool use (page visits, time on each page, time to complete the tool, differential attention to content by sociodemographics), and satisfaction with the information presented in both the intervention and control conditions.

## Discussion

Many consumers apply for help with certified application counselors or navigators to facilitate insurance decisions. However, our prior work demonstrated that even highly educated, trained application counselors struggle to simplify the complexity of health insurance plan choices. Decision support tools should be employed to help present users with options that they can understand, tailored to their needs and preferences. These tools can be used prior to meeting with an application counselor, during a visit with an application counselor, or after meeting with an application counselor.

Several tools are already being developed, including some developed by private companies, some state-specific tools based on the federal “insurance checkbook,” and a new out-of-pocket cost calculator available on healthcare.gov. Our tool, Show Me My Health Plans, goes further than these tools by pulling in data from the Medical Expenditure Panel Survey (MEPS) and creating a tailoring algorithm to estimate yearly out-of-pocket expenditures personalized to individuals and families. Our code and algorithm will be open source so that others can use it and modify it as appropriate.

We will work closely with state level groups (e.g., the Cover MO Coalition, MO Health Net, Missouri Foundation for Health, Health Literacy Missouri) to disseminate our findings. Stakeholders (certified application counselors, policy makers, uninsured participants) will be asked both open-ended and closed-ended questions in order to gather feedback about delivering the tool in community settings beyond the duration of this proposal to ensure broader applicability of our tool. We will develop electronic communication strategies and host webinars to facilitate rapid dissemination [24] and maximize the usefulness of these findings in ongoing policy discussions.

This study can identify particular characteristics of individuals or groups that might face problems accessing and understanding information in the health insurance marketplace. They would therefore be less likely to take up health insurance, creating the possibility of adverse selection in health insurance markets. The findings from this study can lead to specific suggestions on ways to make health care information accessible and informative to the previously uninsured.

## Abbreviations

ACA: Affordable Care Act
ADHD: attention deficit hyperactivity disorder
COPD: chronic obstructive pulmonary disease
FPL: federal poverty level
MEPS: Medical Expenditure Panel Survey
